# Trafficking-Deficient G572R-hERG and E637K-hERG Activate Stress and Clearance Pathways in Endoplasmic Reticulum

**DOI:** 10.1371/journal.pone.0029885

**Published:** 2012-01-05

**Authors:** Ying Wang, Xiaoyan Huang, Jianqing Zhou, Xi Yang, Di Li, Haiyan Mao, Huan Huan Sun, Ningsheng Liu, Jiangfang Lian

**Affiliations:** 1 Ningbo Medical Center, LiHuiLi Hospital School of Medicine, Ningbo University, Ningbo, China; 2 Department of Surgery, University of Rochester Medical Center, Rochester, New York, United States of America; 3 Department of Pathology, Nanjing Medical University, Nanjing, China; Institute of Molecular and Cell Biology, Singapore

## Abstract

**Background:**

Long QT syndrome type 2 (LQT2) is the second most common type of all long QT syndromes. It is well-known that trafficking deficient mutant human *ether-a-go-go-related* gene (hERG) proteins are often involved in LQT2. Cells respond to misfolded and trafficking-deficient proteins by eliciting the unfolded protein response (UPR) and Activating Transcription Factor (ATF6) has been identified as a key regulator of the mammalian UPR. In this study, we investigated the role of ER chaperone proteins (Calnexin and Calreticulin) in the processing of G572R-hERG and E637K-hERG mutant proteins.

**Methods:**

pcDNA3-WT-hERG, pcDNA3-G572R-hERG and pcDNA3-E637K-hERG plasmids were transfected into U2OS and HEK293 cells. Confocal microscopy and western blotting were used to analyze subcellular localization and protein expression. Interaction between WT or mutant hERGs and Calnexin/Calreticulin was tested by coimmunoprecipitation. To assess the role of the ubiquitin proteasome pathway in the degradation of mutant hERG proteins, transfected HEK293 cells were treated with proteasome inhibitors and their effects on the steady state protein levels of WT and mutant hERGs were examined.

**Conclusion:**

Our results showed that levels of core-glycosylated immature forms of G572R-hERG and E637K-hERG in association with Calnexin and Calreticulin were higher than that in WT-hERG. Both mutant hERG proteins could activate the UPR by upregulating levels of active ATF6. Furthermore, proteasome inhibition increased the levels of core-glycosylated immature forms of WT and mutant hERGs. In addition, interaction between mutant hERGs and Calnexin/Calreticulin was stronger after proteasome inhibition, compared to WT-hERG. These results suggest that trafficking-deficient G572R-hERG and E637K-hERG mutant proteins can activate ER stress pathways and are targeted to the proteasome for degradation. Calnexin and Calreticulin play important roles in these processes.

## Introduction

Congenital long QT (LQT) syndrome is a heterogeneous genetic disease and LQT type 2 (LQT2) is the second most common type. It is characterized by delayed ventricular repolarization, QT prolongation on ECG, development of ventricular arrhythmias (torsades de pointes) and sudden deaths, particularly in children and teenagers [1]–[3]. To date, twelve genes have been identified to be responsible for LQT syndrome [4]–[6]. The human *ether-a-go-go-related* gene (hERG, also known as KCNH2) gene encodes the Kv11.1 protein α-subunit, which assembles into a voltage-gated K channel on plasma membrane and underlies the rapidly activating delayed rectifier K-current (I_Kr_) in the heart [7]. Mutations in hERG channels have been implicated in the pathophysiology of LQT2 [8], [9]. To date, approximately 300 hERG mutations have been identified in LQT2 patients [10]–[12]. The most common mechanism of hERG channel dysfunction in these patients is defective protein-trafficking resulting in retention in the endoplasmic reticulum (ER) and failure to reach the plasma membrane [13].

Cells respond to the expression of misfolded and trafficking-deficient transmembrane proteins by eliciting the unfolded protein response (UPR), an ER stress pathway that increases the synthesis of chaperones proteins [14], [15]. UPR consists of both translational and transcriptional regulations. Activating Transcription Factor 6 (ATF6) has been identified as a key regulator of transcriptional control in the mammalian UPR [16]. Specifically, UPR activates the cleavage of ATF6 into its activated form, which then upregulates the synthesis of ER chaperone proteins [16], [17]. Molecular chaperones play important roles in the biogenesis and quality control of many proteins, including glycoproteins [18], [19]. Calnexin and Calreticulin are two key chaperone proteins in the ER responsible for ensuring the proper folding of newly synthesized proteins, as well as other quality control mechanisms [14], [15]. Misfolded and trafficking-deficient proteins retained in the ER are eventually degraded by a process termed ER-associated degradation (ERAD). According to current models, ERAD substrates undergo retro-translocation or dislocation from the ER to the cytosol, where they are degraded by the ubiquitination-proteasome pathway [18].

Although hERG channels have been studied extensively, little is known about the exact mechanism underlying the maturation and processing of trafficking-deficient hERG mutant proteins. G572R-hERG and E637K-hERG are two mutant forms of the hERG channel that have been previously reported as trafficking-deficient [20], [21]. However, the exact process by which they are retained in the ER is not well understood. In the present study, we evaluated the role of Calnexin/Calreticulin in the processing of trafficking-deficient G572R-hERG and E637K-hERG mutant proteins. Specifically, we studied whether the ER stress pathway is involved and whether the mutant proteins are degraded by ubiquitin-mediated proteasome. Our results suggest that trafficking-deficient G572R-hERG and E637K-hERG mutant proteins can activate UPR and are targeted to the proteasome for degradation. Furthermore, the interaction between mutant hERG proteins and chaperone proteins, Calnexin and Calreticulin, play crucial roles in these processes.

## Methods

### cDNA cloning and cell culture

The WT-hERG was cloned into pcDNA3 vector (Invitrogen; Carlsbad, CA) as described previously [22]. G572R-hERG and E637K-hERG mutants were generated by site-directed mutagenesis and subcloned into pcDNA3 vector at BstEII/XhoI restriction sites. These constructs were introduced into HEK293 and U2OS cells through transient transfection using Lipofectamine™ 2000 as described by the manufacturer (Invitrogen). Transfection efficiency was estimated to be approximately 90%. HEK293 and U2OS cells were cultured in Dulbecco's modified Eagle's medium, supplemented with 10% fetal bovine serum, maintained in a humidified 5% CO_2_ incubator at 37°C. Cells were not used beyond 30 passages.

### Western blotting analysis

HEK293 cells expressing WT and mutant hERGs in 100-mm-diameter culture dishes were harvested 48 hours after transfection. Proteins were separated on SDS polyacrylamide gels and then transferred to polyvinylidene difluoride membranes (Pierce Biotechnology, USA). Primary antibodies used included rabbit polyclonal anti-hERG (Alomone Labs, Israel), rabbit polyclonal anti-ATF6 (Active Motif, USA), mouse monoclonal anti-Calnexin (Santa Cruz Biotechnologies, USA) and anti-Calreticulin (Abcam, USA). Blots were visualized with SuperSignal West Pico Chemiluminescent Substrate (Pierce Biotechnology, USA) and developed using Syngene Chemi-Genius imaging system (SynGene, UK).

### Immunofluorescence and confocal imaging

U2OS cells seeded on coverslip in 6-well plate were transiently transfected with pcDNA3-WT-hERG, pcDNA3-G572R-hERG or pcDNA3-E637K-hERG plasmid. At 48 hours post-transfection, cells were fixed with paraformaldehyde, permeabilized with 0.1% Triton X-100 and blocked with 5% goat serum at room temperature (RT). Cells were then labeled with rabbit polyclonal anti-hERG (1∶25 dilution) and mouse monoclonal anti-Calnexin or anti-Calreticulin (1∶25 dilution) at 4°C overnight, followed by incubation with FITC-conjugated goat anti-rabbit IgG secondary antibody and TRITC-conjugated goat anti-mouse IgG secondary antibody at RT for 2 hours. Signals were captured with a Leica TCS SP2 confocal laser scanning microscope.

### Coimmunoprecipitation

HEK293 cells were transiently transfected with pcDNA3-WT-hERG, pcDNA3-G572R-hERG or pcDNA3-E637K-hERG plasmid and then lysed in 500 µl of immunoprecipitation buffer (50 mM Tris-HCl, pH 8.0, containing 150 mM NaCl, 1 mM CaCl2, and 1% Triton X-100) with protease inhibitors (100 µM PMSF, 1 µg/ml pepstatin A, 1 µg/ml leupeptin, 4 µl/ml aprotinin). Cell lysates were pre-cleared by incubation with protein G plus-agarose beads (Santa Cruz Biotechnologies, USA) and incubated with 3 µg of antibody against Calnexin or Calreticulin at 4 °C overnight. The antigen-antibody complexes were isolated with protein G plus-agarose beads and washed with the immunoprecipitation buffer. The bound antigens were eluted from the protein G plus-agarose beads by 2×sample buffer and analyzed by immunoblotting with anti-hERG, anti-Calnexin and anti-Calreticulin antibodies.

### Proteasome inhibitor studies

Transiently transfected HEK293 cells were treated with 20 µM lactacystin, 50 µM ALLN or 100 µM leupeptin for 24 hours. Part of the cell lysates were immunoblotted with anti-hERG, anti-Calnexin and anti-Calreticulin antibodies. The remaining lysates were immunoprecipitated with anti-ubiquitin (Santa Cruz Biotechnologies, USA), anti-hERG, anti-Calnexin or anti-Calreticulin antibodies.

### Cycloheximide chase experiments

HEK293 cells were transiently transfected with G572R-hERG and E637K-hERG plasmids. At 24-hrs post- transfection, cells were treated with 100 µg/ml of cycloheximide and harvested at 0, 4, 8, and 12 hr post-treatment.

## Results

### G572R-hERG and E637K-hERG mutants co-localize with Calnexin/Calreticulin in the ER and they result in protein trafficking deficiency

**Figure 1:**
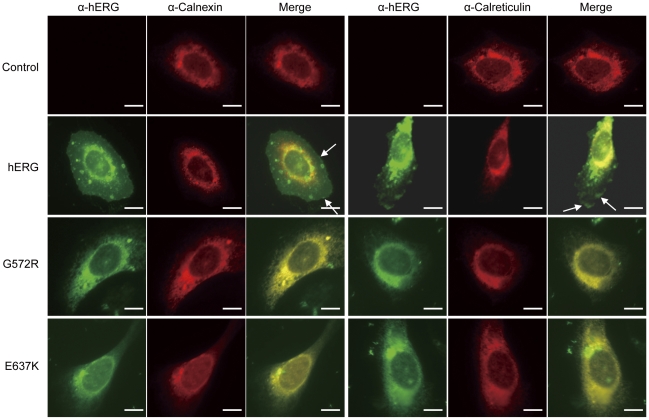
Confocal imaging of WT-hERG and G572R-hERG/E637K-hERG channels in U2OS cells. U2OS cells were transfected with WT-hERG, G572R-hERG and E637K-hERG plasmids and co-stained with anti-hERG (green) and anti-Calnexin or anti-Calreticulin (red) antibodies, as indicated. Calnexin and Calreticulin localize to the ER (top row) while WT-hERG (second row) localizes to both plasma membrane (indicated by white arrows) and cytoplasm. G572R-hERG (third row) and E637K-hERG (bottom row) mutants localize exclusively to the ER, as shown by the overlap with ER markers (Calnexin and Calreticulin) (scale bar 5 µm).

The subcellular localization and protein trafficking of WT-hERG, G572R-hERG and E637K-hERG proteins were examined by immunostaining and confocal imaging (**Fig. 1**). U2OS cells transfected with pcDNA3-WT-hERG, pcDNA3-G572R-hERG and pcDNA3-E637K-hERG plasmids were co-stained with hERG antibody (green; the first and fourth columns) and ER marker proteins - Calnexin antibody (red; the second column) and Calreticulin antibody (red, the fifth column). The confocal images clearly demonstrate that WT-hERG protein is expressed both on the cell surface (arrow) and within the cytoplasm. In contrast, G572R-hERG and E637K-hERG proteins are mainly expressed in the cytoplasm. Specifically, the mutant hERG proteins are retained in the ER with Calnexin and Calreticulin, as suggested by the uniform yellow color in the merged images (the third and sixth columns). Therefore, we conclude that Calnexin/Calreticulin co-localize with G572R-hERG and E637K-hERG proteins in the ER.

**Figure 2:**
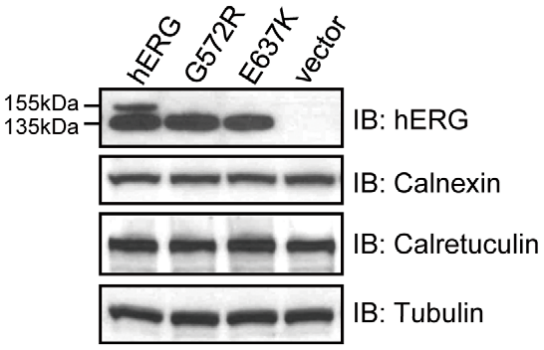
Analysis of WT-hERG, G572R-hERG and E637K-hERG protein expressions in HEK293 cells. Lysates from HEK293 cells expressing WT-hERG, G572R-hERG or E637K-hERG were immunoblotted with anti-hERG antibody. Only WT-hERG expressing cells have both the fully-glycosylated, mature form and core-glycosylated, immature form of the hERG protein. Both G572R-hERG and E637K-hERG expressing cells only have the core-gylcosylated, immature forms of the hERG protein.

To better understand the maturation and trafficking of wild-type and mutant hERG proteins, HEK293 cells were transiently transfected with pcDNA3-WT-hERG, pcDNA3-G572R-hERG or pcDNA3-E637K-hERG plasmid and analyzed by Western blot. In **Figure 2**, WT-hERG transfected cells (first lane) show two protein bands with the upper band at 155 kDa representing the fully-glycosylated, mature form of the protein transported to the cell membrane surface. The lower band at 135 kDa represents the core-glycosylated, immature form of the protein localized to the ER. In contrast, G572R-hERG and E637K-hERG transfected cells (second and third lanes) express only the immature form of the protein at 135 kDa but no fully-glycosylated form of the protein of 155 kDa. These data suggest that the G572R-hERG and E637K-hERG mutant proteins are trafficking-deficient such that only the core-glycosylated, immature form of the protein is expressed.

### Calnexin/Calreticulin interact with core-glycosylated forms of WT-hERG, G572R-hERG and E637K-hERG

**Figure 3:**
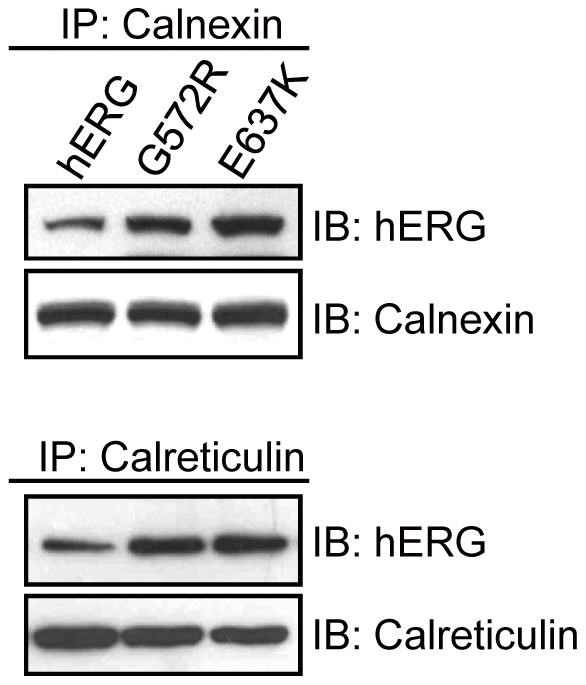
Association of hERG channels with Calnexin and Calreticulin. Lysates from HEK293 cells expressing WT-hERG, G572R-hERG and E637K-hERG were immunoprecipitated with anti-Calnexin and anti-Calreticulin antibody and immunoblotted with anti-hERG antibody. The association of Calnexin/Calreticulin with the core-glycosylated, immature forms of G572R-hERG and E637K-hERG is much stronger than that with WT-hERG.

To assess the physical interaction between hERG and Calnexin/Calreticulin, immunoprecipitation was done with anti-Calnexin or anti-Calreticulin antibody, followed by Western blot analysis with anti-hERG antibody. As shown in **Figure 3**, the core-glycosylated, immature forms of WT-hERG, G572R-hERG and E637K-hERG all interact with Calnexin/Calreticulin while the fully-glycosylated, mature forms of hERG proteins do not. Moreover, the proportion of G572R-hERG or E637K-hERG associated with Calnexin/Calreticulin is greater in comparison with WT-hERG, indicating that G572R-hERG and E637K-hERG have prolonged associations with Calnexin/Calreticulin.

### G572R-hERG and E637K-hERG activate ER stress responses via activation of ATF6

**Figure 4:**
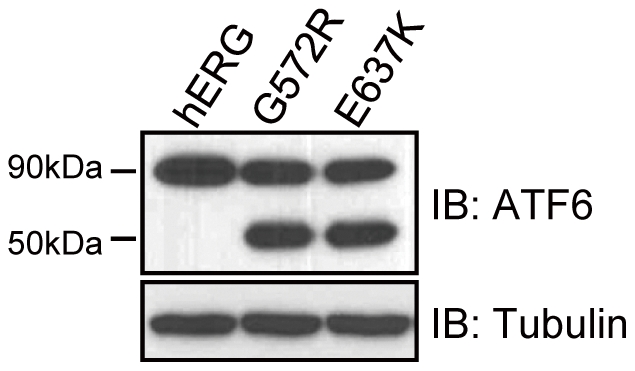
Activation of ATF6 by G572R-hERG and E637K-hERG. HEK293 cells were transfected with WT-hERG, G572R-hERG or E637K-hERG plasmid. Cell lysates were subjected to Western blotting with anti-ATF6 antibody. The cleaved, activated form of ATF6 at 50 kDa is only detected in G572R-hERG and E637K-hERG expressing cells but not in WT-hERG expressing cells.

Since Calnexin/Calreticulin are chaperone proteins with important regulatory roles in the ER quality control pathways, we want to test whether the trafficking-deficient G572R-hERG and E637K-hERG mutants can activate ER stress responses. Given that ATF6 has been identified as a key regulator of transcriptional control during the mammalian UPR, HEK293 cells were transfected with wild-type and mutant hERG protein plasmids and ATF6 protein expression was analyzed by Western blot. The cells were harvested and lysed at 48-hrs post-transfection. As shown in **Figure 4**, WT-hERG, G572R-hERG and E637K-hERG expressing cells all have an upper band at 90 kDa, which represents the unprocessed ATF6 embedded in ER. However, only G572R-hERG and E637K-hERG expressing cells (second and third lanes) have a lower band at 50 kDa, which represents the cleaved form of ATF6 capable of activating transcription of ER stress response genes. These results indicate that expression of G572R-hERG and E637K-hERG mutants result in accumulation of folding-deficient proteins in the ER, which in turn elicits the UPR, as demonstrated by activation of ATF6.

### Mutant hERG channels are degraded through ubiquitin-mediated proteasome pathway

**Figure 5:**
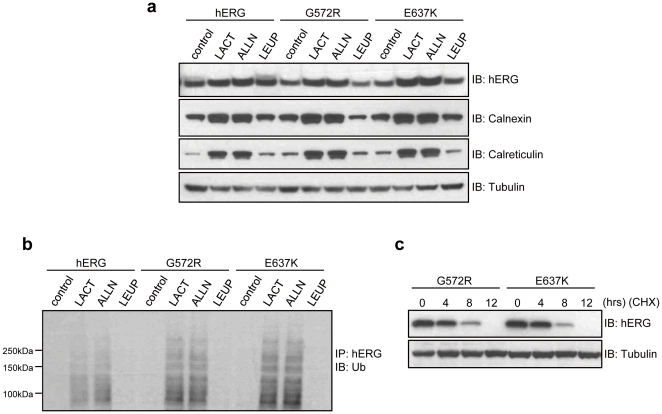
Degradation of G572R-hERG and E637K-hERG mutant channels through ubiquitin-proteasome pathway. **a**, HEK293 cells were transfected with WT-hERG, G572R-hERG or E637K-hERG plasmid and then treated with 20 uM lactacystin (LACT), 50 uM ALLN or 100 uM leupeptin (LEUP) for 24 hours. The cell lysates were subjected to Western blotting with anti-hERG, anti-Calnexin and anti-Calreticulin antibodies. Proteasome inhibition leads to an increase in the core-glycosylated, immature forms of hERG protein with a more significant increase seen in G572R-hERG and E637K-hERG expressing cells in comparison with WT-hERG expressing cells. **b**, Transfected HEK293 cells were treated with proteasome inhibitors or leupeptin as indicated. Lysates were immunoprecipitated with anti-hERG antibody and immunoblotted with anti-ubiquitin antibody. Proteasome inhibition results in the accumulation of poly-ubiquitinated G572R-hERG/E637K-hERG and immature WT-hERG (135 kDa). **c**, Transfected HEK293 cells were treated with cycloheximide (CHX) and harvested at indicated time points. Lysates were immunoblotted with anti-hERG antibody. Protein levels of both G572R-hERG and E637K-hERG mutants are decreased after cycloheximide treatment with half-life less than 6 hrs.

To assess the involvement of proteasome-mediated degradation of hERG proteins, we examined the effects of proteasome inhibitors (lactacystin and ALLN) on the steady state protein levels of WT-hERG, G572R-hERG and E637K-hERG. As shown in **Figure 5a** **,** treatment with lactacystin or ALLN but not leupeptin (a lysosome inhibitor) results in an increase in the levels of core-glycosylated, immature form of hERG, Calnexin and Calreticulin. To further validate that the immature forms of wild-type and mutant hERG proteins are degraded through the ubiquitination-mediated proteasome pathway, transiently transfected HEK293 cells were treated with lactacystin, ALLN or leupeptin and lysates were subjected to immunoprecipitation with anti-hERG antibody. Precipitates were then immunoblotted with antibody against poly-ubiquitinated proteins. We found that proteasome inhibition increases levels of hERG poly-ubiquitination and to a greater extent for G572R-hERG or E637K-hERG than WT-hERG (**Fig. 5b**). However, treatment with leupeptin had no effect at all on hERG poly-ubiquitination. Next, we proceeded to examine the rate of hERG mutant degradation. Protein translation in G572R-hERG and E637K-hERG expressing cells was inhibited by cycloheximide treatment and hERG protein levels were analyzed at the indicated time points. As shown in **Figure 5c**, protein levels of both G572R-hERG and E637K-hERG mutants are decreased after cycloheximide treatment with half-life less than 6 hrs.

**Figure 6:**
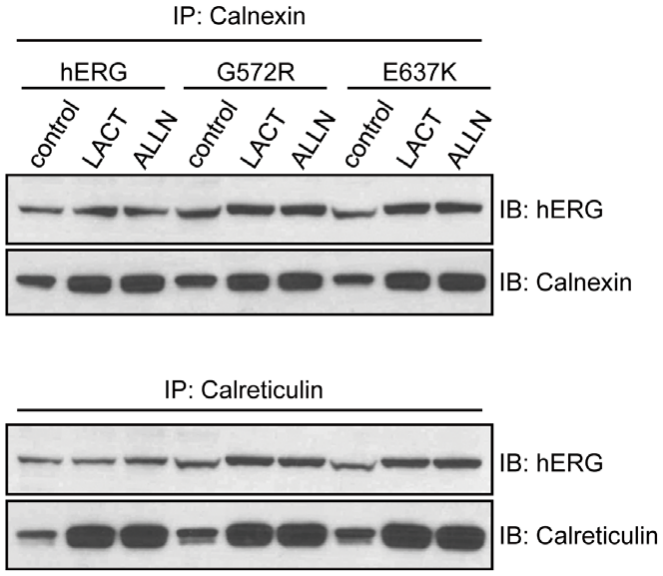
Proteasome inhibition increases association of G572R-hERG/E637K-hERG with Calnexin/Calreticulin. HEK293 cells were transfected with WT-hERG, G572R-hERG or E637K-hERG plasmid. Cells were then treated with proteasome inhibitors and immunoprecipitated with anti-Calnexin or anti-Calreticulin antibody and immunoblotted with anti-hERG antibody. In comparison to WT-hERG, treatment with either LACT or ALLN results in an increase in binding of Calnexin/Calreticulin to G572R-hERG or E637K-hERG.

To further examine the role of proteasome in the degradation of hERG proteins, coimmunoprecipitation analysis was performed. Similarly, transiently transfected HEK293 cells were treated with lactacystin or ALLN and cells were harvested at 24-hours post-treatment. The cell lysates were then immunoprecipitated with anti-Calnexin or anti-Calreticulin antibody and the precipitates were immunoblotted with anti-hERG antibody. As shown in **Figure 6**, the interaction between Calnexin/Calreticulin and hERG mutant proteins has increased significantly after lactacystin and ALLN treatment. However, proteasome inhibitor treatment has a much weaker effect on the association of Calnexin/Calreticulin with the immature form of hERG protein in WT-hERG expressing cells, as shown in **Figure 6**. Furthermore, there was no interaction between the mature form of hERG protein and Calnexin/Calreticulin.

## Discussion

Previous studies have shown that misfolded, incompletely folded and unassembled proteins are retained in the ER. This process is under stringent surveillance by the ER quality control system, which involves molecular chaperones that transiently associate with newly synthesized proteins to promote their proper folding and assembly [13], [19]. Misfolded and unassembled proteins undergo retro-translocation or dislocation from the ER to the cytosol, where they are degraded by the proteasome [18]. Calnexin and Calreticulin are chaperone proteins in the ER that play important roles in the quality control pathways. G572R-hERG and E637K-hERG are mutant hERG proteins that have been previously reported as trafficking-deficient and our current study seeks to elucidate the mechanism by which they are processed in the ER [20], [21]. 

Our results show that the trafficking-deficient G572R-hERG and E637K-hERG mutant proteins are mainly expressed in the cytoplasm and retained in the ER in association with Calnexin/Calreticulin. Furthermore, the proportion of Calnexin/Calreticulin in association with the core-glycoyslated, immature forms of G572R-hERG and E637K-hERG mutants is higher than that for WT-hERG. This indicates that the hERG mutant proteins fail to fold properly and thereby, accumulate in the ER in prolonged association with Calnexin/Calreticulin. Similarly, Gong et al has shown that the N470D-hERG mutant results in protein folding-deficiency and has prolonged association with Calnexin but not Calreticulin [23]. It has also been reported that when arginine vasopressin V2 receptor is mutated, Calnexin has a prolonged association with it [24]. These results collectively illustrate that the chaperone complex functions to arrest mutant proteins in the ER and attempts to re-fold them into the correct native conformation.

It is well-established that the ER quality control system operates efficiently such that only correctly folded molecules can exit the ER while misfolded proteins accumulate in the ER and activate the UPR [25]. In our current study, baseline comparison done in HEK293 cells expressing wild-type and mutant hERG proteins show that the cleaved, activated form of ATF6 is only detected in G572R-hERG and E637K-hERG expressing cells but not in WT-hERG expressing cells. This suggests that an elevated stress response is found in mutant hERG expressing cells, which is consistent with previous report of ATF6 activation by the I593R-hERG mutant [23]. In turn, activated ATF6 upregulates expression of chaperone proteins. Ficker et al has demonstrated the importance of cytosolic chaperones such as Hsp90 and Hsp70 in the maturation of wild-type hERG protein as well as the retention of trafficking-deficient hERG mutants [26]. Our results show that the mutant hERG proteins accumulate in the ER and thereby, activate the UPR. Specifically, expression of G572R-hERG and E637K-hERG mutant proteins result in an increase in the synthesis of chaperones proteins via activation of ATF6.

Misfolded proteins retained in the ER are degraded by the ERAD process, which targets its substrates for ubiquitin-mediated proteasome degradation pathway. After treatment with proteasome inhibitors (lactacystin and ALLN), our results show that there is a notable increase in levels of core-glycosylated, immature forms of G572R-hERG and E637K-hERG proteins as well as accumulation of poly-ubiquitinated mutant hERG proteins. Furthermore, proteasome inhibition increases the association of Calnexin/Calreticulin with G572R-hERG and E637K-hERG. Previous study has also shown that lactacystin or ALLN treatment results in the accumulation of poly-ubiquitinated Y611H-hERG proteins in the ER and prolonged association with molecular chaperone compared to WT-hERG [10]. Collectively, these results strongly suggest that the ubiquitin-mediated proteasome pathway plays an important role in the ER retention and degradation of immature forms of G572R-hERG and E637K-hERG mutant proteins.

In conclusion, our findings provide evidence that the trafficking-deficient E637K-hERG and G572R-hERG mutants are retained in the ER and targeted to the proteasome for degradation. In addition, Calnexin/Calreticulin have prolonged association with E637K-hERG and G572R-hERG mutant proteins and may contribute to their degradation. Therefore, elucidating the exact mechanism by which Calnexin/Calreticulin interact with the mutant hERG channels will provide further insights into the pathophysiology underlying LQT2 and also facilitate the search for new pharmacological methods to restore proper trafficking of mutant hERG channels.
